# Crystallized and fluid intelligence are predicted by microstructure of specific white‐matter tracts

**DOI:** 10.1002/hbm.24848

**Published:** 2019-11-05

**Authors:** Daylín Góngora, Mayrim Vega‐Hernández, Marjan Jahanshahi, Pedro A. Valdés‐Sosa, Maria L. Bringas‐Vega, CHBMP

**Affiliations:** ^1^ The Clinical Hospital of Chengdu Brain Science Institute, MOE Key Laboratory for Neuroinformation University of Electronic Science and Technology of China Chengdu China; ^2^ Cuban Neuroscience Center Havana Cuba; ^3^ UCL Queen Square Institute of Neurology London UK; ^4^ Ministry of Science, Technology and Environment of Cuba Havana Cuba; ^5^ Ministry of Public Health of Republic of Cuba Havana Cuba

**Keywords:** crystallized intelligence, fluid intelligence, fractional anisotropy, MIMIC model, white‐matter tracts

## Abstract

Studies of the neural basis of intelligence have focused on comparing brain imaging variables with global scales instead of the cognitive domains integrating these scales or quotients. Here, the relation between mean tract‐based fractional anisotropy (mTBFA) and intelligence indices was explored. Deterministic tractography was performed using a regions of interest approach for 10 white‐matter fascicles along which the mTBFA was calculated. The study sample included 83 healthy individuals from the second wave of the Cuban Human Brain Mapping Project, whose WAIS‐III intelligence quotients and indices were obtained. Inspired by the “Watershed model” of intelligence, we employed a regularized hierarchical Multiple Indicator, Multiple Causes model (MIMIC), to assess the association of mTBFA with intelligence scores, as mediated by latent variables summarizing the indices. Regularized MIMIC, used due to the limited sample size, selected relevant mTBFA by means of an elastic net penalty and achieved good fits to the data. Two latent variables were necessary to describe the indices: Fluid intelligence (Perceptual Organization and Processing Speed indices) and Crystallized Intelligence (Verbal Comprehension and Working Memory indices). Regularized MIMIC revealed effects of the forceps minor tract on crystallized intelligence and of the superior longitudinal fasciculus on fluid intelligence. The model also detected the significant effect of age on both latent variables.

## 1 INTRODUCTION

Intelligence has been defined in many ways over the years, leading to still intense controversy in psychology. Definitions have ranged from the operational statement that “intelligence is what Intelligence Quotient (IQ) tests measure” to the proposal of a latent variable reflecting a very general capability that, among other things, involves the ability to reason, plan, solve problems, think abstractly, understand complex ideas, learn quickly, and learn from experience (Gottfredson, 1997). In any case, irrespective of the definition, measures of intelligence have strong correlations with brain imaging and genetic measures (Deary, Penke, & Johnson, 2010), prompting the use of several neuroimaging techniques to try to understand the neural basis of intelligence. The relationship between IQ and brain structure has been explored using many techniques including voxel‐based morphometry, cortical thickness, spectroscopy, and diffusion‐weighted imaging (DWI) (Basten, Hilger, & Fiebach, 2015; Haier, 2009; Joshi et al., 2011; Reiss, Abrams, Singer, Ross, & Denckla, 1996; Yu et al., 2008). These studies suggest that structural and functional brain imaging can express individual differences in brain pathways, particularly the parietofrontal structures (Jung & Haier, 2007) which correlate positively with intelligence (Deary et al., 2010).

An interesting strand of these studies, one that we will follow in this article, suggests links of white‐matter microstructural properties (such as fractional anisotropy or FA) to cognitive information‐processing speed and thus to the neural foundation of general intelligence (Penke et al., 2012). FA quantifies the dispersion of water molecules, and it is constrained by the organization of white‐matter structures. While sometimes interpreted as a measure of white‐matter integrity, FA is a very complex and indirect measure with various limitations, and its relationship to white‐matter health is not yet fully understood (Bender, Prindle, Brandmaier, & Raz, 2016; Jones, Knösche, & Turner, 2013). Nevertheless, FA is widely used, as it has been shown to be associated with individual differences in a range of cognitive domains, especially in old age (Madden et al., 2009). As an example, there are significant correlations between water diffusion parameters and intelligence across the life span, from childhood (Deary et al., 2010) to old adulthood (Charlton et al., 2006; Deary et al., 2006). Other studies have provided evidence that all measures of information‐processing speed, as well as a general speed factor composed from these tests (g‐speed), were significantly associated with FA (Kuznetsova et al., 2015).

Many of these studies suffer from three main shortcomings:

1. The relation of neuroimaging with intelligence measures is usually based on simple correlations. While useful in exploratory analyses, they do not provide information about causal pathways that require more complex multivariate analyses. Specific statistical methods for causal analysis must be used.
2. There is an inconsistent exploration of cognitive domains. Some studies rely on specially designed cognitive scales, then summarized to reflect a single latent variable. Even when widely available scales, such as the Wechsler Adult Intelligence Scale (WAIS) (Wechsler, Sierra, & Blanca, 2003), are used, they are often also reduced to a single score, such as the general or “g” factor (Spearman, 1904), or “fluid intelligence” using different procedures. It is better to explore the individual indices of the WAIS, those related to performance intelligence: Perceptual Organization (PO) and Processing Speed (PS), as well as verbal Intelligence: Verbal Comprehension (VC) and Working Memory (WM).
3. The anatomical accuracy in the definition of the tracts has been variable across studies. Some examples with progressive accuracy are as follows:
  - Initial studies using voxel‐based averages of FA values without close matching to the tract they belonged to voxel based fractional anisotropy.
  - The mean FA values over each tract, where these are defined by the projection fasciculi from a population‐averaged tractography atlas to the subject's native space (mean tract‐based fractional anisotropy [mTBFA]‐atlas).
  - FA averages along tracts obtained from each subject's DWI in native space (mTBFA‐individual).

We now review the solution to these three issues and analyze recent papers which dealt with them.

Regarding the first problem about statistical issues, appropriate multivariate methods to determine causal pathways are now emerging. Specifically, the Structural Equation Modeling (SEM) framework has proven to be particularly useful (Bollen & Hoyle, 2012). These models estimate a set of regression equations which may be interpreted in terms of a directed Bayesian network in which the variables studied are considered as nodes of a graph with each valid regression equation a directed edge. This graph fulfills a Markov property that can be explained as follows: If variable A is connected to B and B to C, and there is no other direct or indirect path from A to C, B “totally mediates” the influence of A on C. In other words, B “screens off” A from C. Under appropriate conditions (Pearl, 2000), the resulting graphs allow some inference about mechanistic causal relations. For a discussion of these concepts in brain networks, see Valdes‐Sosa, Roebroeck, Daunizeau, and Friston (2011). A particularly useful type of SEM is the Multiple Indicator, Multiple Causes (MIMIC) introduced by Jöreskog and Goldberger (2006) in which latent variables are introduced as mediators between two sets of observed variables. This framework was leveraged by Kievit et al. (2012, 2016) and Kievit, Fuhrmann, Borgeest, Simpson‐Kent, and Henson (2018) to provide an SEM specification for the study of the relation of FA with intelligence, work that is worth summarizing in the next paragraph.

In Kievit et al. (2012), a MIMIC model was estimated with the following three levels to the data of 80 subjects: (a) Voxel‐based region of interest (ROI) measures for four FA (VBFA) tracts and 4 Gy matter, (b) a single g (WAIS) latent variable, (c) the four WAIS indices (WM, VC, PO, and PS). This model was applied and showed a good fit but considered only a single cognitive domain based on a sample with a limited age range (18–29 years) and a limited small number of FA measures that were not actually tract based.

The former study was followed by a seminal paper by Kievit et al. (2016) that embodied statistically the “watershed model” proposed by Cannon and Keller (2006) within the MIMIC framework. That model postulated that multiple causes act through latent variables (endophenotypes) to produce the observable phenotypes. This MIMIC/watershed model was fit to a cross‐sectional sample of 555 subjects aged from 18 to 87 years of age (Cam‐CAN; Shafto et al., 2014). The model comprised four levels, the first level consisting of 10 mBTFA‐atlas measures (Table 1). The second level was six speed of processing (CAM/CAN). The third level was a unique latent variable identified with “fluid intelligence” (FI‐CAM/CAM). Hereon we will identify psychometric scales and latent variable by the initials of the study. Finally, the last level consisted of the four subtests of fluid intelligence of Cattell's Culture Fair, Scale 2, Form A (Cattell, 1971). This model was built up by a successive exploration of increasingly more complex models verified with confirmatory factor analyses. The overall model gave an excellent fit to such a large sample, verifying the use of the MIMIC approach. Note that age was not included as an explicit variable at level one.

**Table 1:** White‐matter tracts included in the analysis

Full name of white‐matter tract	Abbreviation
Anterior thalamic radiation	ATR
Cingulum associated to cingulate gyrus	CGC
Cingulum associated to hippocampal gyrus	CGH
Corticospinal tract	CST
Forceps major	Fmj
Forceps minor	Fmn
Inferior fronto‐occipital fasciculus	IFO
Inferior longitudinal fasciculus	ILF
Superior longitudinal fasciculus	SLF
Uncinate fasciculus	UNC

Subsequent to the Kievit et al. (2016) proposal of the watershed model for intelligence regularized regression methods were integrated into SEM/MIMIC modeling (Jacobucci, Brandmaier, & Kievit, 2019). Regularization (Zou & Hastie, 2005) imposes restrictions on the relations between coefficients allowing the exploration of large sets of variables with built‐in selection of those that are relevant. This model was used to study subjects from the UK Biobank (Kievit et al., 2018) in a longitudinal study with three waves, with 185,317, 9,719, and 870 subjects, respectively. As acknowledged by the authors, the cognitive domains explored were limited by the UK Biobank design with a conflation of fluid and crystal intelligence items. Interestingly, a three‐level MIMIC model was integrated with a longitudinal one to analyze individual variable trajectories. This time, a three‐level model was assumed:

1. Fifteen mTBFA individual for a set that includes those described in Table 1;
2. Coefficients of the age regression (two latent variables);
3. Fluid intelligence scores (FI‐UK Biobank).

In this very large longitudinal study, the use of MIMIC models is once more validated, the effect of aging is discussed, and the use of personalized (mTBFA‐individual) and not atlas‐based FA measures was introduced. As the authors state, the “suboptimal task design” restricted the exploration of cognitive interpretation. Besides, findings from the same group (Kievit et al., 2018) showed a weak but significant negative association between age and fluid intelligence.

Our study reported here will attempt to fill in some of the gaps of previous studies. We will take advantage of one of the rare population‐based studies based in a country in Latin America: the second wave of the Cuban Human Brain Mapping Project (CHBMP; Hernandez‐Gonzalez et al., 2011). This cross‐sectional study evaluated more than 95% of a random sample of 2,109 subjects with medical, cognitive, and neuroimaging studies (*T*1, *T*2, DWI, electroencephalogram (EEG)) to yield a final group of 240 “functionally healthy” subjects, of which 83 (aged 18–69 years) were used in this study. Not only does this allow us to reexamine some of the unanswered questions from previous studies but also to construct an MIMIC (watershed model) that integrates previous approaches by the following:

1. Exploring a wider range of cognitive domains namely, the four WAIS indices for intelligence as in Kievit et al. (2012).
2. Partitioning for the first time, the variance of these indices among the optimal number of latent variables.
3. Using individualized average FA tract‐based measures of white‐matter microstructure as in Kievit et al. (2018).
4. Constructing a watershed model of the influence of white‐matter microstructure, mediated by latent factors, on the full set of indices of the WAIS.

## 2 MATERIAL AND METHODS

### 2.1 Participants

The sample included 83 healthy right‐handed participants with an average age of 35.03 ± 10.27 years and 12.12 ± 2.46 years of education. The recruitment was based on a completely randomized sampling using the identity card database stratified by age, gender, and outward ethnic features of 2,109 subjects of the whole population of La Lisa municipality (more than 30,000) in La Habana. It is important to note that this municipality was selected because its demography closely matched those of the general Cuban population according to the national census of the Republic of Cuba: http://www.one.cu/.

The present study was carried out in accordance with The Code of Ethics of the World Medical Association, Declaration of Helsinki (World Medical Organization, 2013), and the experimental protocols were approved by the Ethics Committee of the Cuban Neuroscience Center. The recruitment procedure did not involve any kind of reward, but only feedback about the results and participants were included in the study after accepting and signing the informed consent.

### 2.2 Assessments

Each participant underwent an interview and medical examination with specialists in Neurology and Psychiatry, in order to rule out chronic diseases (e.g., addictions, including smoking) or any disorders of the nervous system that would invalidate their participation in the study. Neurological examination was performed following the procedure described in the guidelines published by the U.S. Department of Health and Human Services in 2003 (Neurological Single System Examination in http://www.cms.gov/MLNEdWebGuide/25_EMDOC.asp). The Mini‐International Psychiatric Interview was used for psychiatric evaluation (Sheehan et al., 1998). Intelligence was assessed using the fully validated and translated to Spanish language version of the WAIS‐III (Wechsler et al., 2003), printed and distributed in Mexico by The Manual Moderno (https://www.worldcat.org/title/wais-iii-escala-weschler-de-inteligencia-para-adultos-iii/oclc/54053545). This scale provided scores for a Full Scale IQ (FSIQ), Verbal IQ (VIQ), and Performance IQ (PIQ) along with four secondary indices: PO, PS, VC, and WM. The subtests included in each index were as follows: PO: picture completion, block design, matrix reasoning; PS: digit‐symbol coding and symbol search; VC: vocabulary, similarities, information, comprehension; and WM: arithmetic, digit span, letter‐number sequencing. The raw measures were scored according to the official normative data included in the printed version of WAIS‐III. However, to avoid culture bias, they were subsequently standardized with information from the Cuban sample to produce scores of the specific performance, adjusted for age for our population.

### 2.3 Magnetic resonance imaging acquisition protocol

A Siemens 1.5 T Magnetom Symphony system with a standard birdcage head coil for signal transmission/reception (Siemens, Erlangen, Germany) was used to acquire images, including a high‐resolution *T*1‐weighted anatomical image and a standard diffusion sequence.

The *T*1‐weighted structural image (1 × 1 × 1 mm resolution) was acquired with the following parameters: echo time (TE) = 3.93 ms, repetition time (TR) = 3,000 ms, flip angle = 8°, and field of view (FOV) = 256 × 256. This yielded 160 contiguous 1‐mm‐thick slices in a sagittal orientation. Axial diffusion weighted images were acquired along 12 independent directions, in 50 slices spaced at 3 mm, with 2 × 2 mm in‐plane resolution, and a diffusion weighting *b* value of 1,200 s/mm^2^. The following parameters were used: FOV = 128 × 128, TE = 160 ms, TR = 7,000 ms, flip angle = 90°. A reference image (*b*0 image) with no diffusion weighting was also obtained (*b* = 0 s/mm^2^).

In order to correct the distortions caused by magnetic field inhomogeneities in the series of diffusion‐weighted images, phase and magnitude maps were obtained. The parameters used were voxel size of 3.5 mm, ET_1_ = 7.71 ms, ET_2_ = 12.47 ms, and RT = 672 ms. The Diffusion Tensor Imaging images were movement, eddy‐current, and distortion corrected. Using the magnitude and phase images and the unwarping functionality (Anderson, 2001), the effects of the principal inhomogeneities of magnetic fields were corrected. Later, the diffusion tensor and the FA were determined in each voxel (Pierpaoli & Basser, 1996).

### 2.4 Fiber tracking computation

Computation of the diffusion tensor and fiber tracking was performed using DTI&FiberTools v.3.0 (http://www.uniklinik-reiburg.de/mr/live/arbeitsgruppen/diffusion_en.html; Kreher, Hennig, & Il'yasov, 2006) and implemented in Matlab (The MathWorks, 2014). According to the formulation of Basser, Mattiello, and LeBihan (1994), and by diagonalizing the diffusion tensor for each voxel, the toolbox generates as output six components of a diffusion tensor, three eigenvectors that characterize the direction of diffusion, and three eigenvalues that characterize the magnitude of the diffusion in the corresponding eigenvector calculated (Basser et al., 1994).

Three‐dimensional reconstruction of the tracts was performed using the deterministic tractography method Fiber Assignment by Continuous Tracking algorithm and a brute‐force reconstruction approach (Mori, Crain, Chacko, & Van Zijl, 1999). Fiber tracking was initiated by specifying six parameters: the minimum FA threshold for starting tracking, the minimum FA for stopping tracking, the maximum trace (Tr) for starting tracking, the maximum trace for stopping tracking, the critical angle threshold for stopping tracking in case the algorithm encounters a sharp turn in the fiber direction, and a minimum fiber length. The start criteria used in the reconstruction of the tracts were FA = 0.15, Tr = 0.0016, and a stop criteria FA = 0.10, Tr = 0.002. A turning angle threshold of 53.1° and minimum fiber length of five voxels were used. The DTI&Fiber Tools v.3.0 used these parameters to generate the coordinates of all fibers in the brain from which the tract trajectory is reconstructed after drawing an ROI in a user‐defined region of the brain.

### 2.5 Definition of tract‐based fractional anisotropy (mTBFA)

A multiple ROIs approach was used for the reconstruction of the tracts of interest because it has been shown that the two‐ROI and brute‐force approaches could effectively reduce the sensitivity to the noise and ROI placement (Huang, Zhang, van Zijl, & Mori, 2004). The fiber tracking was performed on every voxel of the brain, and fibers that penetrated the previously defined ROIs were assigned to the specific tracts associated with each pair of ROIs.

Definition of ROIs for studied tracts was made by replicating a set of predefined ROI by Mori et al. (2002) that was employed successfully in subsequent work (Góngora, Domínguez, & Bobes, 2016; Mori et al., 2008; Wakana et al., 2007; Wakana, Jiang, Nagae‐Poetscher, Van Zijl, & Mori, 2004). The following procedure replicated the methodology published by Góngora et al. (2016). These ROIs were drawn using the program MRIcron (http://www.mricron.com) on a reference anatomical image with a spatial resolution of 1 × 1 × 1 mm^3^ in stereotactic space of the Montreal Neurological Institute (Evans et al., 1993). The ROIs were then transformed to each individual brain space automatically using the SPM toolbox functionalities (Friston, Ashburner, Kiebel, Nichols, & Penny, 2007). In this routine, the high‐resolution anatomical *T*1 image was realigned to the standard position on the AC–PC plane and normalized using the procedures of SPM. The unnormalized *T*1 was rigidly co‐registered with the *b*0 image using a mutual information cost function (Collignon et al., 1995). The ROIs were defined for the following tracts defined in Table 1. The resulting path of these tracts was visually inspected and corrected in cases where necessary by the exclusion of fibers that did not belong anatomically to tracts. The mean tract‐based FA (mTBFA) was obtained as an estimate of the average along each tract, which resulted from the superposition of the specific coordinates for each tract on the corresponding maps of FA. For the statistical analysis, this parameter was averaged between the corresponding bilateral tracts.

### 2.6 Statistical analysis

In the present study, we first used the MIMIC model to determine the number of latent variables necessary to explain the four indices of WAIS‐III. For this purpose, we first fitted a single latent g (WAIS‐III) model as in Kievit et al. (2012) as a composite of PO and PS, and VC and WM. Subsequently, a two‐latent variable model was fitted to these same indices corresponding to verbal and performance intelligence scales.

Subsequently, the sample was fitted with a modification of the watershed model proposed by Kievit et al. (2016). In our model, anatomical variables (mTBFA) affect the two intelligence latent variables that in turn mediate the WAIS‐III indices. Age is also included as affecting the latent variables as in Kievit et al. (2018). Exploratory analysis regarding possible confounds (gender, handedness, and educational level) in the estimations was performed but without relevant effects in the estimations and will not be further considered in this article.

Estimates may be imprecise when using maximum likelihood estimation with large numbers of variables and a limited sample size. We therefore used the regularized SEM model (Jacobucci et al., 2019) implemented in the *cv_regsem* function (*regsem* package in R) (Jacobucci, Grimm, & McArdle, 2016) as a postprocessing of a *laavan* model. We used an Elastic‐net regularization (equal proportion of Lasso and Ridge regularization). The regularization parameter *λ*, which determines the number of variables to keep in the model, was explored across 35 values of *λ* ranging from 0 to 0.35. The elastic‐net method provides a compromise between sparsity and variable selection of cluster of related variables (Zou & Hastie, 2005). To choose a final model among the 35 models run, the Bayesian information criterion (BIC; Schwartz, 1978) was used, which approximates the Bayesian model evidence, thus providing a trade‐off metric of model fit and model complexity in which the best model achieves the lowest value. The final model is shown as that selected by the regularized SEM package.

## 3 RESULTS

Analysis of the IQ measures

**Figure 1:**
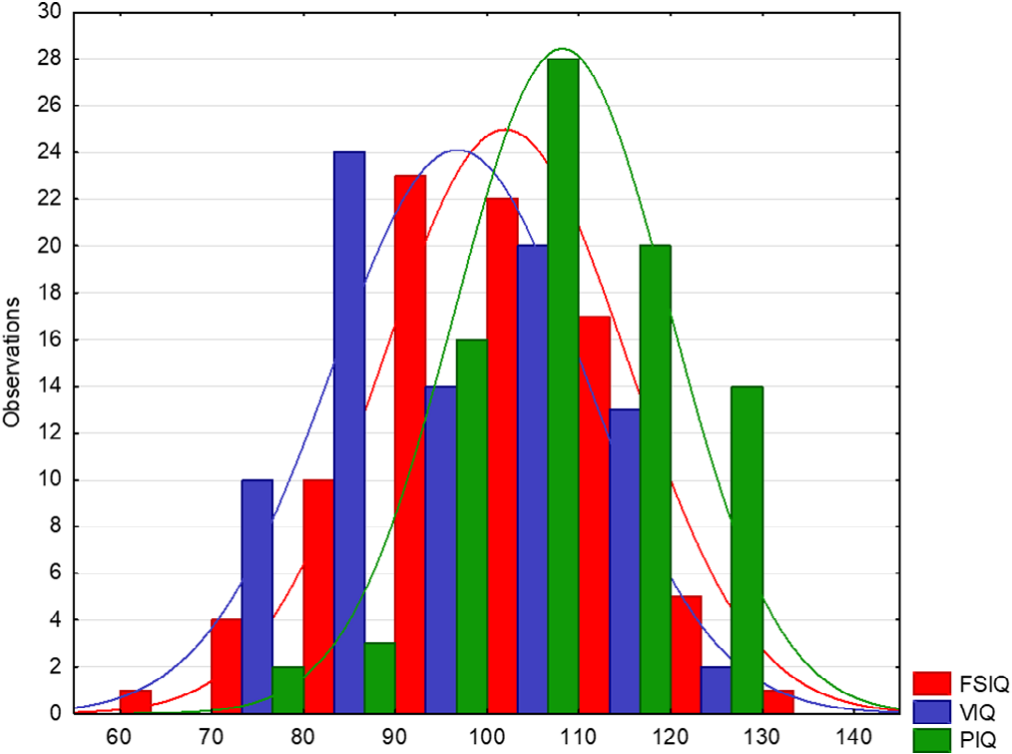
Histogram of Wechsler Adult Intelligence Scale III (Wechsler et al., 2003). FSIQ, Full Scale Intelligence Quotient; PIQ, Performance Intelligence Quotient; VIQ, Verbal Intelligence Quotient

The application of the WAIS‐III resulted in the estimation of a mean FSIQ of 101.75 ± 13.25, a mean VIQ of 96.58 ± 13.73, and a mean PIQ of 107.94 ± 11.64, which were in the average range and were normally distributed (Figure 1). The means for the four indices were PO: 27.71 ± 6.77, PS: 16.63 ± 4.97, VC: 29.39 ± 7.42, and WM: 26.17 ± 5.77.

As a first step for the full MIMIC model, we first built up the measurement model using only the WAIS‐III indices. We tested the adequacy of the single latent variable g‐WAIS. This model showed an almost acceptable, but not good, fit, with a χ^2^ = 5.589, *df* = 2, *p* = .061, Root Mean Square Error of Approximation (RMSEA) = 0.161 (0.000–0.326), Comparative Fix Index (CFI) = 0.963, Standardised Root Mean Square Residual (SRMR) = 0.044, and Satorra–Bentler scaling factor = 1.196. The BIC of this model was 831.075. All the indexes contribute significantly to their corresponding latent variables with a *p* < .001.

We subsequently fitted a second model for the indices, now considering two latent variables: one corresponding to the verbal (VC and WM) and the other to the performance (PO and PS)‐related indices. This model also fits the data very well: χ^2^ = 0.189, *df* = 1, *p* = .664, RMSEA = 0.000 (0.000–0.226), CFI = 1, SRMR = 0.008, and Satorra–Bentler scaling factor = 1.045. The BIC of this model was lower than the single latent variable one with an estimate of 825.854 indicating a better fit. Note that all the indices contributed significantly to their corresponding latent variables with a *p* < .001. For the rest of the article, we used these two latent factors identifying the verbal one with “crystallized intelligence” and the performance one with “fluid intelligence” according to Cattell (1963)), denoted in this article as CI and FI without any further clarification. The detailed breakdown of the contribution of the indices to latent factors is as follows. For FI, PO has an *R* ^2^ of 0.856 while PS: 0.408. For CI, the *R* ^2^ of VC is 0.756 and WM 0.483.

### 3.1 MIMIC (watershed) model of integrated WM and cognitive measures

We fitted a regularized SEM/MIMIC/Watershed model with an elastic‐net penalty (Jacobucci et al., 2019), introducing the following hierarchical levels:

1. The most upstream variables were the 10 major white‐matter tract mTBFA, which were modeled as influencing only the two latent variables. Note that age was also added at this level as in Kievit et al. (2018).
2. A next level where the two latent variables obtained in the previous section: Fluid and Crystallized intelligence.
3. Finally, at the lowest level, the four WAIS‐III indices (VC, PO, WM, and PS) mediated by the latent variable of their corresponding cognitive domain.

**Figure 2:**
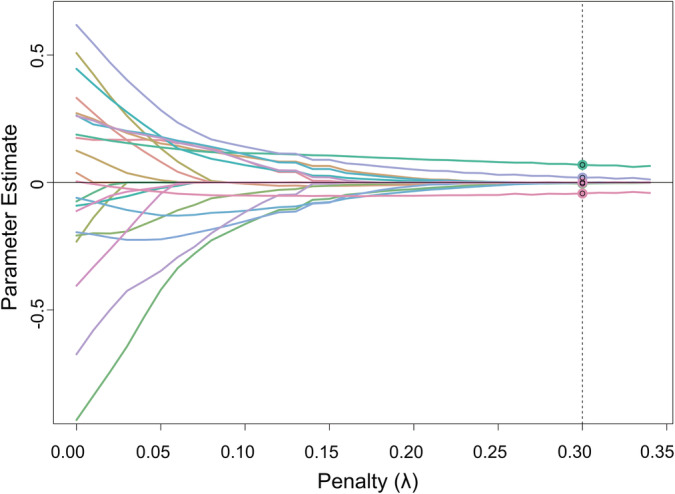
Parameter trajectory plot from regularized MIMIC. The graph shows the values of the regression coefficients as a function of the penalty value. The dashed vertical line highlights the penalty value yielding the model with the best fit (i.e., the lowest Bayesian information criterion)

Note that for this implementation of regularized SEM, *p* values are not estimated. Instead, there is a selection of regression coefficients (or edges in the SEM graph) thus finessing the need for multiple comparisons. The parameter trajectory plot of this model is shown in Figure 2, which shows the value of the coefficients for each value of the regularization parameter which controls the trade‐off between model fit and the penalty imposed. The elastic net imposes a balance (in our case, half and half) between an *L*_1_ sparseness penalty and an *L*_2_ ridge penalty (see Jacobucci et al. 2019 and for a more detailed discussion Valdes‐Sosa et al., 2005) for a discussion of this and other similar models in the detection of neural networks.

**Figure 3:**
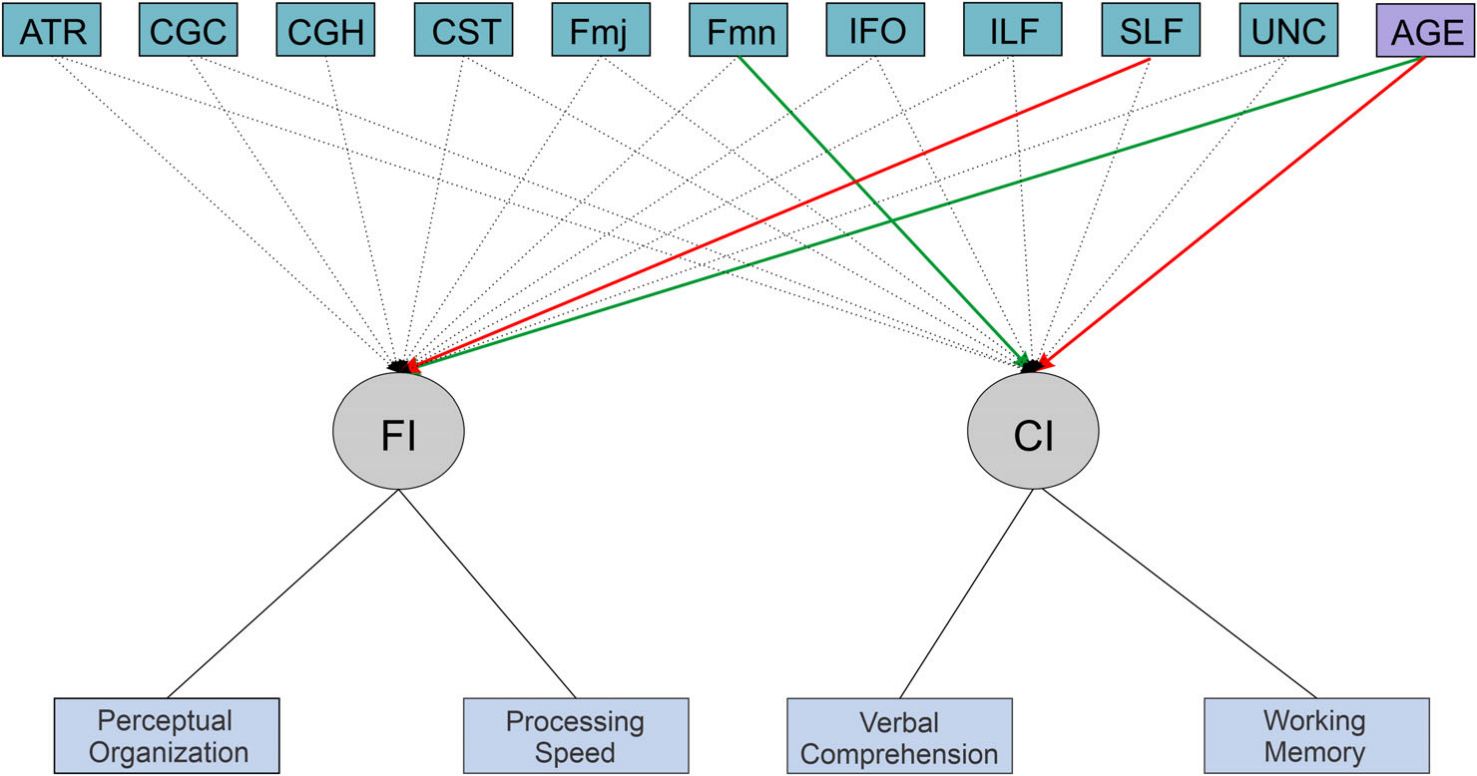
A regularized MIMIC model of the relationship between two latent variables (CI and FI) and white‐matter tracts where nonzero model estimates edges are by solid lines and the color indicates whether the effect is positive (green) or negative (red). The zero estimates edges are represented by dotted lines. CI, Crystallized Intelligence; FI, Fluid intelligence; MIMIC, Multiple Indicators, Multiple Causes

The value of the regularization parameter lambda selected was 0.3 corresponding to the lowest BIC value of 0.836. The resulting model is depicted in Figure 3. The latent variables are represented as circles in the resulting diagrams and the observed variables as squares (Schreiber, Stage, King, Nora, & Barlow, 2006). From Figure 3, it can be seen that the edges selected by the regularized MIMIC (with the coefficient values in parenthesis) were as follows:

1. Age to FI (0.069) and CI (−0.043)
2. Forceps Minor (Fmn) to CI (0.019)
3. Superior longitudinal fasciculus (SLF) to FI (−0.004)

As noted in Jacobucci et al. (2019), this type of variable selection may not be familiar to some researchers who are more used to *p* values or *R*‐squared statistics.

## 4 DISCUSSION

### 4.1 Structure of cognitive variables

Previous studies using MIMIC have postulated a single latent variable mediating between FA and cognitive indexes. In fact, there are many studies, focusing only on relations between intelligence indices, that have demonstrated that rather than a single general factor, there are several relatively independent factors needed to explain intelligence in terms of different cognitive domains. Particularly important is the confirmatory factor analysis of a sample of 6,832 individuals from 33 cross‐sectional studies where five correlated first‐order factors were identified: reasoning, spatial ability, memory, PS, and vocabulary (Salthouse, 2004), which are very closely related but not identical to the four independent indexes of the WAIS‐III: PO or Reasoning (PO), PS, VC, and WM. Thus, the single‐factor summarization of intelligence scales in Kievit et al. (2016, Kievit et al., 2018) lead to a misallocation of variance of the intelligence measures that will affect the full model.

By contrast, here we leveraged the availability of the full WAIS‐III recorded with DWI from the CHBMP. Our analysis showed that two latent factors, fluid intelligence or FI and crystalized intelligence or CI, provide better fits than a single general or g (WAIS) latent variable. This is in agreement with the observation of Kievit et al. (2016) who suggested that fitting more than one latent variable, with the exploration of more cognitive domains, would increase the explained variance of the models.

### 4.2 Full MIMIC model relating white‐matter microstructure to intelligence indexes, mediated by latent variables

As mentioned before, the model produced by *regsem* is not accompanied by *p* values or confidence intervals, which may seem surprising. Jacobucci et al. (2019) point out that this type of model incurs acceptable bias when using regularization, but the more important aim is the holdout sample generalization, which is achieved by reducing variance and preferring models of a complexity that is afforded by the observed data. In this framework, the resulting edges left in the MIMIC model are important if they are simply not set to zero. This is the interpretation we will follow here.

The regularized MIMIC model indicated an age effect on both FI and CI. This points to the effectiveness of this method to unravel more subtle relations in a set of predictors since age was not significant in nonregularized versions of MIMIC models. This method of regularization is supported by previous studies that showed its ability to model the relation between cognitive performance and imaging metrics, taking a high‐dimensional set of predictors and reducing this set to create a relatively parsimonious representation of key tracts previously implicated in specific tasks, for example, visual short‐term memory performance (Jacobucci et al., 2019).

The regularized MIMIC model revealed two tracts that are connected directly to the latent variables. We describe these two paths of putative causation next.

One tract, Fmn is an interhemispheric tract whose fibers connect left and right frontal lobes through the genu of the corpus callosum (Mori, Wakana, Van Zijl, & Nagae‐Poetscher, 2005), the enhanced anatomical connectivity of which may underlie the greater fluid reasoning, visuospatial WM, and creative capabilities appreciated in mathematically gifted children (Navas‐Sánchez et al., 2014). The microstructural characteristics of interhemispheric connections have been positively correlated to some intelligence variables in females but negatively correlated in males (Tang et al., 2010).

In our model, the Fmn was connected to the CI latent variable, therefore also predicting performance in the WM and VC indices. In contradistinction, a positive association of the Fmn with the FI (Cam‐CAN) has been reported by Kievit et al. (2016). This is not surprising since FI (Cam‐CAN) in the words of the authors they are all subtests of a single cognitive domain (Cattell, 1971). Even more complicated is the comparison with results of the UK Biobank study (Kievit et al., 2018) since the latent variable is defined with a set of measures that have been suggested to be difficult to interpret (Lyall et al., 2016).

Our model also identified another white‐matter tract as important, the SLF. The SLF is located over the cingulum running from the dorsal and medial parietal cortex to premotor and prefrontal cortices (Schmahmann et al., 2007). Specifically, it is on the superior lateral portion of the putamen forming a long arch that emits branches toward the temporal, parietal, and occipital lobes (Mori et al., 2005). This tract connects the caudal part of the inferior parietal lobule and intraparietal sulcus, areas that are involved in visuospatial information processing. Also, their fibers get to the posterior prefrontal cortex, which is of great importance in perception and awareness (Petrides & Pandya, 2006; Schmahmann et al., 2007).

In our model, the SLF influences FI with a negative sign, which in turn influences PS and PO. This negative path between SLF and the latent variable FI suggests that those subjects with higher FI scores will have lower FA values. This result, at first sight, is counterintuitive if FA reflects “white‐matter integrity” or “increased speed” of neural activity along the corresponding fiber tracts. Similar lack of concordance with the “myelin integrity” hypothesis was found by Braddick et al. (2017) for the correlation of individual children's sensitivity to global motion coherence with the FA of the left SLF. Hoeft et al. (2007) also showed increased FA of SLF was associated with poor visuospatial abilities in Williams' syndrome, which argues against the white‐matter FA identification. In addition, the negative relation between the FA of the SLF and memory tests has been previously reported (Tang et al., 2010), at least for males in the left branch of the SLF.

This apparently counterintuitive result may be viewed as we stated in the introduction, due to the fact that FA is a highly complex measure that must be interpreted with caution (Jones et al., 2013). Although it is true that FA is directly related to increased myelin thickness, parallelism, and packing of axons, it also depends on other factors such as barriers and obstacles imposed by microstructure, cell membranes, myelin sheaths, and microtubules (Beaulieu, 2002). It may decrease with larger axonal diameters due to an increase in the mobility of water in the intra‐axonal compartment water mobility (Takahashi et al., 2002). Fiber crossings may be another factor influencing the observed values of FA. This may explain both positive and negative correlations of FA with reaction time as reported and discussed by Tuch et al. (2005). A deeper understanding of white‐matter microstructural determinants of cognitive functions will require improved diffusion magnetic resonance imaging (MRI) technology and methods (Jelescu & Budde, 2017; Jones et al., 2013; Riffert, Schreiber, Anwander, & Knösche, 2014).

Interestingly, the effect of the SLF was restricted to FI while we were expecting some influence over CI. There are some reports of a relationship of this white‐matter tract with the tests which composed CI. For example, a multiple sclerosis study by combining the Paced Visual Serial Addition Test with functional MRI‐guided fiber tractography found the SLF was the main white‐matter tract connecting areas active during this attention and WM task (Bonzano, Pardini, Mancardi, Pizzorno, & Roccatagliata, 2009). In addtition, Papagno et al. (2017) found that direct electrical stimulation of the SLF during awake surgery improves verbal short‐term memory. This also supports the participation of the SLF in the so‐called “phonological loop,” which has been described as a crucial component for language acquisition (Papagno et al., 2017).

### 4.3 Limitations and future work

Several limitations should be noted in the present research which pertain to study design, measures of brain organization, and statistical methodology. We detail them here and point out that the next wave of the CHBMP will take them into consideration.

The sample is relatively small with data gathered cross‐sectionally. A larger sample is foreseen for the third wave of the CHBMP, and collaborative studies with other Latin American countries are being organized. A major difficulty to perform combined analyses with other databases from the United States or Europe is the lack of harmonization of their cognitive studies. There are also the following problems related to measures of brain organization:

1. Even when the trajectories obtained agreed with neuro‐anatomic descriptions derived from postmortem and other in vivo tractography studies (Carpenter & Sutin, 1983; Góngora et al., 2016; Mori et al., 2002; Nieuwenhuys, Voogd, & Van Huijzen, 2007; Wakana et al., 2007), the existence of inaccuracies due to partial volume effects, noise, and crossing fibers involve the visualized pathways do not necessarily reflect brain connectivity since individual axons could be merging and blanching at any point along the bundle (Wakana et al., 2007).
2. A further limitation is related to the quality of DWI images gathered in the second wave of the CHBMP:
  - Only 12 diffusion‐sensitizing gradient directions were recorded (too sparse to allow higher order diffusion models);
  - Also, only limited voxel size was possible (2 × 2 × 3) that could affect the robustness to noise, partial volume effect, and crossing fiber regions. These limitations were due to the technology available in 2004 in Cuba, which has been since then modernized.
3. The fiber tracking algorithm (Fiber Assignment by Continuous Tracking) employed is highly susceptible to errors in the orientation of the principal eigenvector, due both to noise and to instances where the direction of the underlying tract anatomy is ambiguous, for example, assessment of voxels where fiber bundles cross, diverge, or converge. Improved algorithms now exist and will be used in the future.
4. The only measure of brain organization analyzed is FA, which as we have discussed previously is not very specific. Improved measures of white‐matter microstructure are to be preferred (Riffert et al., 2014)
5. Instead of exploring only the effects of white‐matter differences on individual intelligence, it would be preferable to use a model based on integrating model of DWI, functional Magnetic Resonance Imaging (fMRI), and possibly EEG as previously described (Valdés‐Sosa, Vega‐Hernández, Sánchez‐Bornot, Martínez‐Montes, & Bobes, 2009). This type of causal neural connectivity model (Valdes‐Sosa et al., 2011) might then be used as the upstream construct in an extended MIMIC model of intelligence.

Despite these limitations, we believe this study is valuable due to the completely randomized sampling of participants from the general Cuban population, the personalized determination of tract‐based FA analysis, and the administration of the complete WAIS‐III. In fact, one of the limitations of previous work was the limited measures of FI employed (four subtests of fluid reasoning) collected on the Cam‐CAN project (Shafto et al., 2014). This issue was overcome in our study employing more WAIS‐III measures, opening a wider spectrum of cognitive domains, and finding other significant associations. On the other hand, the selection of two latent variables, independent of the evaluator seems to be a reasonable alternative to reduce the random or systematic measurement errors associated with observable variables (IQ scores). The MIMIC model revealed the effect of distinct fiber tracts, Fmn and SFL, on FI and CI, respectively. Work to overcome the limitations and extend this line of research will be pursued in the future.

## 5 CONCLUSIONS

The present work was inspired by the watershed model of Kievit et al. (2016) who proposed the use of a MIMIC hierarchical model, using a regularized SEM version. We found this approach very useful. By exploring the full set of indices of the WAIS‐III, it was possible to find novel associations of FA with four cognitive domains mediated by latent variables relating to both fluid intelligence (SLF) and crystallized intelligence (Fmn). The relation of the Fmn tract with crystalized intelligence was only discoverable by including in the model indices related to this domain, something that seems to have been overlooked in many studies. In fact, we believe that a wide range of cognitive functions could be explored, something apparently not yet envisaged in the current large brain projects.

## CONFLICT OF INTERESTS

The authors declare that the research was conducted in the absence of any commercial or financial relationships that could be construed as a potential conflict of interest.

## AUTHOR CONTRIBUTIONS

M.L.B. conceived this and directed this study as part of the Cuban Human Brain Mapping Project which was coordinated by P.V.S. D.G. performed the DTI data processing, methodology, analysis, software implementation, and writing of the first manuscript under the supervision of M.L.B. and P.V. M.V. contributed in the statistical analysis. M.J. contributed to the final revision and editing of this article.

## Supporting information


**Table S1** Cognitive test only model estimates from Watershed model including only one latent variable (g factor) set from the cognitive indexes.

**Table S2** Model estimates from MIMIC which included two latent variables set from the cognitive tests and regressions among structural variables (FA measures) and including the age as additional regressor.

## Data Availability

Imaging data is available at http://cbmp-ccc.cneuro.cu. The dataset employed here, including behavioral assessment and tractography estimations, is available at doi: 10.6084/m9.figshare.8959529.
