# Supplementary material for: Crystallized and fluid intelligence are predicted by microstructure of specific white‐matter tracts
Source: Hum Brain Mapp. 2019 Nov 5;41(4):906–16. doi: 10.1002/hbm.24848 (PMC7267934; doi:10.1002/hbm.24848)
Supplement: Supplementary file 1 — Table S1 Cognitive test only model estimates from Watershed model including only one latent variable (g factor) set from the cognitive indexes. [file HBM-41-906-s001.docx]

Supplemental Material.

Table 1. Cognitive test only model estimates from Watershed model including only one latent variable (g factor) set from the cognitive indexes.

Optimization method NLMINB

Number of free parameters 8

Number of observations 83

Estimator ML Robust

Model Fit Test Statistic 6.683 5.589

Degrees of freedom 2 2

P-value (Chi-square) 0.035 0.061

Scaling correction factor 1.196

for the Satorra-Bentler correction

Model test baseline model:

Minimum Function Test Statistic 123.876 109.467

Degrees of freedom 6 6

P-value 0.000 0.000

User model versus baseline model:

Comparative Fit Index (CFI) 0.960 0.965

Tucker-Lewis Index (TLI) 0.881 0.896

Robust Comparative Fit Index (CFI) 0.963

Robust Tucker-Lewis Index (TLI) 0.890

Loglikelihood and Information Criteria:

Loglikelihood user model (H0) -410.479 -410.479

Loglikelihood unrestricted model (H1) -407.138 -407.138

Number of free parameters 8 8

Akaike (AIC) 836.958 836.958

Bayesian (BIC) 856.309 856.309

Sample-size adjusted Bayesian (BIC) 831.075 831.075

Root Mean Square Error of Approximation:

RMSEA 0.168 0.147

90 Percent Confidence Interval 0.038 0.316 0.000 0.285

P-value RMSEA <= 0.05 0.062 0.087

Robust RMSEA 0.161

90 Percent Confidence Interval 0.000 0.326

Standardized Root Mean Square Residual:

SRMR 0.044 0.044

Parameter Estimates:

Information Expected

Information saturated (h1) model Structured

Standard Errors Robust.sem

Latent Variables:

Estimate Std.Err z-value P(>|z|) Std.lv Std.all

g =~

Prcptl_Orgnztn 0.828 0.103 8.063 0.000 0.828 0.833

Processing_Spd 0.623 0.102 6.120 0.000 0.623 0.626

Verbl_Cmprhnsn 0.793 0.083 9.555 0.000 0.793 0.798

Working_Memory 0.660 0.099 6.683 0.000 0.660 0.664

Variances:

Estimate Std.Err z-value P(>|z|) Std.lv Std.all

.Prcptl_Orgnztn 0.302 0.090 3.362 0.001 0.302 0.305

.Processing_Spd 0.600 0.090 6.706 0.000 0.600 0.608

.Verbl_Cmprhnsn 0.359 0.083 4.302 0.000 0.359 0.363

.Working_Memory 0.553 0.096 5.754 0.000 0.553 0.559

g 1.000 1.000 1.000

R-Square:

Estimate

Prcptl_Orgnztn 0.695

Processing_Spd 0.392

Verbl_Cmprhnsn 0.637

Working_Memory 0.441
