# Supplementary material for: Crystallized and fluid intelligence are predicted by microstructure of specific white‐matter tracts
Source: Hum Brain Mapp. 2019 Nov 5;41(4):906–16. doi: 10.1002/hbm.24848 (PMC7267934; doi:10.1002/hbm.24848)
Supplement: Supplementary file 2 — Table S2 Model estimates from MIMIC which included two latent variables set from the cognitive tests and regressions among structural variables (FA measures) and including the age as additional regressor. [file HBM-41-906-s002.docx]

Supplemental Material.

Table 2. Model estimates from MIMIC which included two latent variables set from the cognitive tests and regressions among structural variables (FA measures) and including the age as additional regressor.

Optimization method NLMINB

Number of free parameters 31

Number of observations 83

Estimator ML

Model Fit Test Statistic 22.603

Degrees of freedom 23

P-value (Chi-square) 0.484

Model test baseline model:

Minimum Function Test Statistic 169.581

Degrees of freedom 50

P-value 0.000

User model versus baseline model:

Comparative Fit Index (CFI) 1.000

Tucker-Lewis Index (TLI) 1.007

Loglikelihood and Information Criteria:

Loglikelihood user model (H0) -395.586

Loglikelihood unrestricted model (H1) -384.285

Number of free parameters 31

Akaike (AIC) 853.173

Bayesian (BIC) 928.157

Sample-size adjusted Bayesian (BIC) 830.375

Root Mean Square Error of Approximation:

RMSEA 0.000

90 Percent Confidence Interval 0.000 0.088

P-value RMSEA <= 0.05 0.721

Standardized Root Mean Square Residual:

SRMR 0.029

Parameter Estimates:

Information Expected

Information saturated (h1) model Structured

Standard Errors Standard

Latent Variables:

Estimate Std.Err z-value P(>|z|) Std.lv Std.all

FI =~

Prcptl_Orgnztn 1.000 0.886 0.891

Processing_Spd 0.744 0.126 5.904 0.000 0.659 0.663

CI =~

Verbl_Cmprhnsn 1.000 0.782 0.787

Working_Memory 0.975 0.145 6.730 0.000 0.763 0.768

Regressions:

Estimate Std.Err z-value P(>|z|) Std.lv Std.all

FI ~

ATR 0.175 0.344 0.508 0.611 0.197 0.196

CGC 0.332 0.286 1.160 0.246 0.375 0.373

CGH 0.038 0.297 0.127 0.899 0.043 0.042

CST 0.272 0.268 1.014 0.311 0.307 0.305

Fmj 0.125 0.311 0.401 0.688 0.141 0.140

Fmn 0.508 0.363 1.399 0.162 0.573 0.569

IFO -0.232 0.520 -0.445 0.656 -0.262 -0.260

ILF -0.208 0.394 -0.529 0.597 -0.235 -0.234

SLF -0.932 0.420 -2.219 0.026 -1.052 -1.046

UNC -0.074 0.273 -0.271 0.786 -0.083 -0.083

Age 0.188 0.119 1.581 0.114 0.212 0.211

CI ~

ATR -0.091 0.314 -0.291 0.771 -0.117 -0.116

CGC 0.446 0.263 1.694 0.090 0.570 0.567

CGH 0.263 0.271 0.969 0.333 0.336 0.334

CST -0.060 0.245 -0.245 0.807 -0.077 -0.076

Fmj -0.195 0.284 -0.686 0.493 -0.249 -0.248

Fmn 0.618 0.334 1.851 0.064 0.790 0.785

IFO -0.674 0.477 -1.411 0.158 -0.861 -0.856

ILF 0.261 0.360 0.726 0.468 0.334 0.332

SLF -0.405 0.384 -1.055 0.292 -0.518 -0.515

UNC -0.112 0.249 -0.448 0.654 -0.143 -0.142

Age 0.003 0.108 0.032 0.974 0.004 0.004

Covariances:

Estimate Std.Err z-value P(>|z|) Std.lv Std.all

.FI ~~

.CI 0.537 0.111 4.819 0.000 0.907 0.907

Variances:

Estimate Std.Err z-value P(>|z|) Std.lv Std.all

.Prcptl_Orgnztn 0.203 0.099 2.058 0.040 0.203 0.205

.Processing_Spd 0.554 0.100 5.521 0.000 0.554 0.561

.Verbl_Cmprhnsn 0.376 0.088 4.290 0.000 0.376 0.381

.Working_Memory 0.406 0.089 4.584 0.000 0.406 0.411

.FI 0.684 0.161 4.241 0.000 0.872 0.872

.CI 0.512 0.136 3.763 0.000 0.836 0.836

R-Square:

Estimate

Prcptl_Orgnztn 0.795

Processing_Spd 0.439

Verbl_Cmprhnsn 0.619

Working_Memory 0.589

FI 0.128

CI 0.164
